# Immunohistochemical Expression of Proliferating Cell Nuclear Antigen in Invasive Breast Carcinoma: Associations With Histological Grade and Ki-67 Expression

**DOI:** 10.7759/cureus.116709

**Published:** 2026-09-22

**Authors:** Vikash Kumar, Amresh Kumar, Namita Singh, Vandana, Bipin Kumar

**Affiliations:** 1 Pathology, Aligarh Muslim University, Aligarh, IND; 2 Pathology, Indira Gandhi Institute of Medical Sciences, Patna, IND

**Keywords:** breast carcinoma, histological grade, immunohistochemistry, ki-67, pcna, proliferating cell nuclear antigen

## Abstract

Background

Proliferating cell nuclear antigen (PCNA) is a nuclear protein involved in DNA replication and repair. Although PCNA is widely used experimentally as a proliferation-related marker, its clinicopathological relevance in breast carcinoma is less clearly established than that of Ki-67. This study evaluated whether increasing ordinal PCNA immunoexpression was associated with increasing histological grade.

Methods

This retrospective cross-sectional study identified 409 breast carcinoma cases evaluated at the Department of Pathology, Jawaharlal Nehru Medical College, Aligarh Muslim University, Aligarh, India, from October 2018 to December 2020. The sample size was estimated to detect a correlation of 0.38 between PCNA and Ki-67, with 80% power at a two-sided alpha level of 0.05. Eligible complete-data cases were sampled using proportionate stratified random sampling according to menopausal status; computer-generated random selection without replacement yielded 26 premenopausal and 26 postmenopausal women. PCNA and Ki-67 immunoexpression were recorded as negative, mild (1+), moderate (2+), or intense (3+). The primary PCNA-grade association was evaluated using Fisher-Freeman-Halton Monte Carlo inference, Spearman correlation, the Jonckheere-Terpstra ordered-trend test, and Cramer's V; Ki-67 was a secondary comparator.

Results

The mean age was 49.90 ± 9.85 years, and all 52 patients were women. Grades 1, 2, and 3 comprised six (11.5%), 32 (61.5%), and 14 (26.9%) tumors, respectively. The final PCNA immunoexpression category was mild (1+) in six cases (11.5%), moderate (2+) in six cases (11.5%), and intense (3+) in 40 cases (76.9%). The 3+ PCNA immunoexpression category occurred in one of six grade 1 tumors, 25 of 32 grade 2 tumors, and all 14 grade 3 tumors. PCNA was significantly associated with grade (Fisher-Freeman-Halton Monte Carlo p<0.001; Cramer's V=0.483), with a positive monotonic relationship (Spearman rho=0.476, p<0.001) and ordered trend (Jonckheere-Terpstra Z=3.340, two-sided Monte Carlo p<0.001). PCNA also correlated with Ki-67 category (rho=0.387, p=0.005).

Conclusions

Increasing PCNA immunoexpression was associated with higher histological grade and showed a moderate positive ordinal association with Ki-67 in this cohort. These findings support a clinicopathological relationship between PCNA expression and proliferative morphology but do not establish independent prognostic or treatment-predictive utility. Larger prospectively assembled studies using standardized quantitative scoring and clinical follow-up are required.

## Introduction

Breast cancer remains one of the most frequently diagnosed malignancies worldwide. The Global Cancer Observatory estimated approximately 2.3 million new female breast cancer cases in 2022, underscoring the continuing need for reliable pathological measures of tumor behavior [1]. Histological type, Nottingham grade, tumor size, nodal status, hormone receptor status, and human epidermal growth factor receptor 2 (HER2) remain central to pathological assessment and clinical risk stratification.

Tumor proliferation is an important biological feature of breast carcinoma. Ki-67 is the most commonly used immunohistochemical proliferation marker, but its interpretation is affected by pre-analytical conditions, scoring approach, intratumoral heterogeneity, and interobserver variability [2,3]. The International Ki67 in Breast Cancer Working Group has therefore emphasized standardized visual assessment and cautioned that clinical utility is clearest at extreme values in selected early-stage, estrogen receptor-positive/HER2-negative tumors [4].

Proliferating cell nuclear antigen (PCNA) is a 36-kDa homotrimeric sliding-clamp protein that supports DNA polymerase processivity and participates in DNA replication, repair, chromatin assembly, cell-cycle regulation, and multiple protein interactions [5]. PCNA expression rises during late G1 and S phases but may persist because of its relatively long half-life and role in DNA repair. Consequently, PCNA can reflect proliferative activity without being specific for cells actively progressing through the cell cycle [6]. Breast cancer studies have reported variable relationships between PCNA expression, Ki-67, histological grade, stage, lymph-node status, and clinical outcome [6-10].

Recent literature continues to consider PCNA a potentially complementary proliferation-related marker, although Ki-67 remains more established clinically [2,10]. The primary objective of the present study was to evaluate the association between ordinal PCNA immunoexpression and histological grade in invasive breast carcinoma. The relationships between PCNA and Ki-67 immunoexpression and between Ki-67 and histological grade were evaluated as secondary objectives. Associations of PCNA with other clinicopathological variables were considered exploratory.

## Materials and methods

Study design and setting

This institution-based retrospective cross-sectional study used archived clinicopathological records and formalin-fixed, paraffin-embedded breast carcinoma tissue evaluated in the Department of Pathology, Jawaharlal Nehru Medical College, Aligarh Muslim University, Aligarh, India. The study period was October 2018 to December 2020.

Sample size, sampling, and case selection

The sample size was estimated to detect a correlation between PCNA and Ki-67 immunoexpression. Sullivan et al. reported a correlation coefficient of r=0.38 between PCNA and Ki-67 labeling indices in breast carcinoma [11]. The required sample size was estimated using Fisher's z transformation: $n=\left[\frac{Z_{1-\alpha/2}+Z_{1-\beta}}{0.5\ln\left(\frac{1+r}{1-r}\right)}\right]^2+3$

where $Z_{1-\alpha/2} = 1.96$ represents a two-sided significance level of 5%, $Z_{1-\beta} = 0.84$ represents 80% statistical power, and (r=0.38) is the anticipated correlation coefficient. Thus, $n=\left[\frac{1.96+0.84}{0.5\ln\left(\frac{1+0.38}{1-0.38}\right)}\right]^2+3\approx51.99\approx52$

The calculated minimum sample size was approximately 52 cases. An exact two-sided power calculation based on the noncentral t-distribution demonstrated that 52 cases provided approximately 81.3% power to detect a correlation coefficient of 0.38 at an alpha level of 0.05. Therefore, the final sample size of 52 cases was considered adequate.

A total of 409 breast carcinoma cases were identified during the study period. The eligible sampling frame comprised female breast carcinoma cases with complete clinicopathological information, including histological grade, stage, estrogen receptor (ER), progesterone receptor (PR), and HER2 status, together with adequate archived formalin-fixed, paraffin-embedded tissue for PCNA and Ki-67 immunohistochemistry. Cases with incomplete required information or inadequate archived tissue were excluded before sampling.

Proportionate stratified random sampling was performed using menopausal status as the stratification variable. Premenopausal and postmenopausal women constituted approximately equal proportions of the eligible sampling frame. Each eligible case was assigned a unique serial number within its respective stratum, and cases were selected using computer-generated random numbers without replacement. Proportional allocation, after rounding, yielded 26 premenopausal and 26 postmenopausal women, producing a final analytic cohort of 52 patients. This procedure preserved the approximate menopausal distribution of the eligible sampling frame while ensuring representation of both strata. The final analytic cohort included no male patients.

Histopathological evaluation

Sections approximately 3-5 micrometers thick were prepared from paraffin blocks and stained with hematoxylin and eosin. Tumor types were transcribed from the original histopathology records and harmonized to the terminology used in the present analysis. Histological grade was assigned using the Nottingham modification of the Scarff-Bloom-Richardson grading system, incorporating tubule formation, nuclear pleomorphism, and mitotic count, as originally described by Elston and Ellis [12]. The component scores were summed to classify tumors as grade 1, 2, or 3. The prognostic relevance of Nottingham histological grade in invasive breast carcinoma has subsequently been validated in a large cohort [13]. Triple-negative breast cancer was treated as an immunophenotype defined by the absence of ER, PR, and HER2 expression rather than as a separate histological type.

Immunohistochemistry and scoring

Archived tissue specimens had been formalin-fixed and paraffin-embedded according to standardized departmental procedures. Fresh sections measuring 3-4 µm in thickness were cut from the archived blocks and mounted on poly-L-lysine-coated glass slides. Immunohistochemical staining was performed manually using the UltraVision Quanto HRP-DAB detection system and reagents supplied by Thermo Fisher Scientific (Waltham, MA, United States). A ready-to-use mouse monoclonal anti-PCNA antibody, clone PC10 (catalogue number MS-106-R7), was used. Following deparaffinization and rehydration, heat-induced epitope retrieval was performed in citrate buffer at 95-98°C for 10-20 minutes. Endogenous peroxidase activity was blocked using hydrogen peroxide for 10 minutes, followed by application of protein block for five minutes. The primary antibodies were incubated for one hour at room temperature. The secondary antibody and HRP polymer were each applied for 10 minutes, and immunoreactivity was visualized using 3,3′-diaminobenzidine for five minutes, followed by hematoxylin counterstaining. Tonsil tissue served as the positive control for Ki-67, ductal breast carcinoma tissue served as the positive control for PCNA, and negative-control sections were processed by omitting the primary antibody.

PCNA immunoexpression was assessed using a semiquantitative additive scoring system incorporating the proportion of positively stained tumour nuclei and the intensity of nuclear staining. Only unequivocal nuclear staining in viable invasive tumour cells was considered positive. After an initial low-power examination of the section, representative areas containing viable invasive tumour were identified. Microscope fields were randomly selected from these representative areas without preferential selection of areas showing the highest staining. Using a ×40 objective, all eligible tumour-cell nuclei within successively selected fields were counted until a total of 500 tumour nuclei had been evaluated in each case. Necrotic areas and non-neoplastic cells were excluded. Assessment was performed by a single pathologist without blinding.

The proportion of positively stained tumour nuclei and nuclear staining intensity were each scored from 0 to 3. Staining intensity was judged by comparison with the positive-control slide processed with the staining run. The proportion and intensity scores were added to obtain a composite score ranging from 0 to 6. The composite score was subsequently converted into the final 0-3+ PCNA immunoexpression category according to the scoring algorithm shown in Table 1. These final composite categories were treated as ordinal variables in the statistical analysis.

**Table 1:** Semiquantitative additive scoring algorithm used to determine the final proliferating cell nuclear antigen (PCNA) immunoexpression category. The final PCNA immunoexpression category represents the combined proportion and intensity scores and does not represent staining intensity alone.

Component	Criterion	Score
Proportion of positive tumour nuclei	No positively stained tumour nuclei	0
	>0% to <5%	1
	5%-10%	2
	>10%	3
Nuclear staining intensity	Absent	0
	Mild	1
	Moderate	2
	Intense	3
Final category: proportion score + intensity score	Composite score 0-2: negative	0
	Composite score 3: mild immunoexpression	1+
	Composite score 4-5: moderate immunoexpression	2+
	Composite score 6: intense immunoexpression	3+

Ki-67 immunoexpression was recorded according to the original semiquantitative categories as negative (0), mild (1+), moderate (2+), or intense (3+). Only unequivocal nuclear staining in tumour cells was considered positive. The Ki-67 categories were used as ordinal variables in the secondary analyses.

Study outcomes

The primary relationship for the present analysis was between ordinal PCNA category and histological grade. The secondary relationships were PCNA versus Ki-67 category and Ki-67 versus histological grade. Associations of PCNA with age, menopausal status, stage, ER, PR, HER2, triple-negative phenotype, tumor size, and lymph-node burden were considered exploratory. No recurrence, treatment-response, disease-free survival, or overall-survival endpoint was available; the study was not designed to establish prognostic performance.

Statistical analysis

Categorical variables were summarized as counts and percentages; age was summarized using mean, standard deviation, and range. PCNA, Ki-67, and grade were treated as ordinal variables. The primary monotonic association was measured using Spearman rank correlation, with Kendall's tau-b as a supporting tie-adjusted analysis. The Jonckheere-Terpstra test evaluated ordered increases in proliferation-marker categories across grades. Contingency-table association was evaluated using Fisher-Freeman-Halton inference with 200,000 Monte Carlo samples and 99% Monte Carlo confidence intervals because multiple expected cell counts were below five. Cramer's V quantified categorical effect size; Goodman-Kruskal gamma and Somers' D provided supporting ordinal effect measures. The Kruskal-Wallis test was retained as a sensitivity analysis. Exploratory analyses were interpreted descriptively because of sparse subgroups and multiplicity. Tests were two-sided, and p<0.05 was considered statistically significant. Analyses were performed using IBM SPSS Statistics for Windows, version 27.0 (IBM Corp., Armonk, NY, USA).

Ethics

The Institutional Ethics Committee, Faculty of Medicine, Aligarh Muslim University, approved the study protocol at its meeting held on December 22, 2018. The approval certificate bears D.No. 250/FM and is dated May 11, 2019. The study was conducted in accordance with the approved protocol and applicable institutional ethical requirements.

## Results

Characteristics of the analytic cohort

The 52 patients were women aged 23-75 years, with a mean age of 49.90 ± 9.85 years. The cohort comprised 26 premenopausal women (50.0%) and 26 postmenopausal women (50.0%). Fifty tumors were invasive ductal carcinoma/no special type, one was metaplastic carcinoma, and one was carcinoma with medullary features. Receptor-defined triple-negative phenotype was present in 20 cases, including 18 invasive ductal carcinomas and the two special histological types. Grade 2 was the predominant grade, and 36 cases (69.2%) were stage III (Table 2).

**Table 2:** Clinicopathological characteristics of the 52-case immunohistochemistry cohort ER: estrogen receptor; PR: progesterone receptor; HER2: human epidermal growth factor receptor 2; NST: no special type; SD: standard deviation.

Characteristic	Category	n (%)
Age, years	Mean ± SD (range)	49.90 ± 9.85 (23-75)
Menopausal status	Premenopausal	26 (50.0)
	Postmenopausal	26 (50.0)
Histology	Invasive ductal carcinoma/NST	50 (96.2)
	Metaplastic carcinoma	1 (1.9)
	Carcinoma with medullary features	1 (1.9)
Triple-negative phenotype	Present	20 (38.5)
Histological grade	Grade 1	6 (11.5)
	Grade 2	32 (61.5)
	Grade 3	14 (26.9)
Stage group	I	1 (1.9)
	II	15 (28.8)
	III	36 (69.2)
ER status	Positive	20 (38.5)
PR status	Positive	17 (32.7)
HER2 status	Positive	16 (30.8)

PCNA immunoexpression category and histological grade

The final PCNA immunoexpression category was mild (1+) in six cases, moderate (2+) in six cases, and intense (3+) in 40 cases; no tumor was assigned to the negative category. A graded shift toward the 3+ category was evident: one of six grade 1 tumors, 25 of 32 grade 2 tumors, and all 14 grade 3 tumors were assigned to the 3+ PCNA immunoexpression category (Table 3 and Figure 1). The sparse 3 × 3 table showed a significant overall association on Fisher-Freeman-Halton Monte Carlo analysis (p<0.001; 99% CI, 0.000380-0.000640), with Cramer's V=0.483. PCNA increased monotonically with grade (Spearman rho=0.476, p<0.001; Kendall tau-b=0.438, p<0.001). The Jonckheere-Terpstra test confirmed an ordered increase (Z=3.340; two-sided Monte Carlo p<0.001; 99% CI, 0.000367-0.000623). The Kruskal-Wallis sensitivity analysis was also significant (H=13.724, p=0.001). Supporting ordinal effect estimates were gamma=0.832 and Somers' d=0.519 when grade was treated as dependent (Table 4).

**Table 3:** Proliferating cell nuclear antigen (PCNA) immunoexpression according to the histological grade Percentages are calculated within grade rows. Fisher-Freeman-Halton Monte Carlo p<0.001; Cramer's V=0.483

Histological grade	Mild PCNA (1+)	Moderate PCNA (2+)	Intense PCNA (3+)	Total
Grade 1	1 (16.7%)	4 (66.7%)	1 (16.7%)	6
Grade 2	5 (15.6%)	2 (6.3%)	25 (78.1%)	32
Grade 3	0	0	14 (100%)	14
Total	6 (11.5%)	6 (11.5%)	40 (76.9%)	52

**Figure 1:**
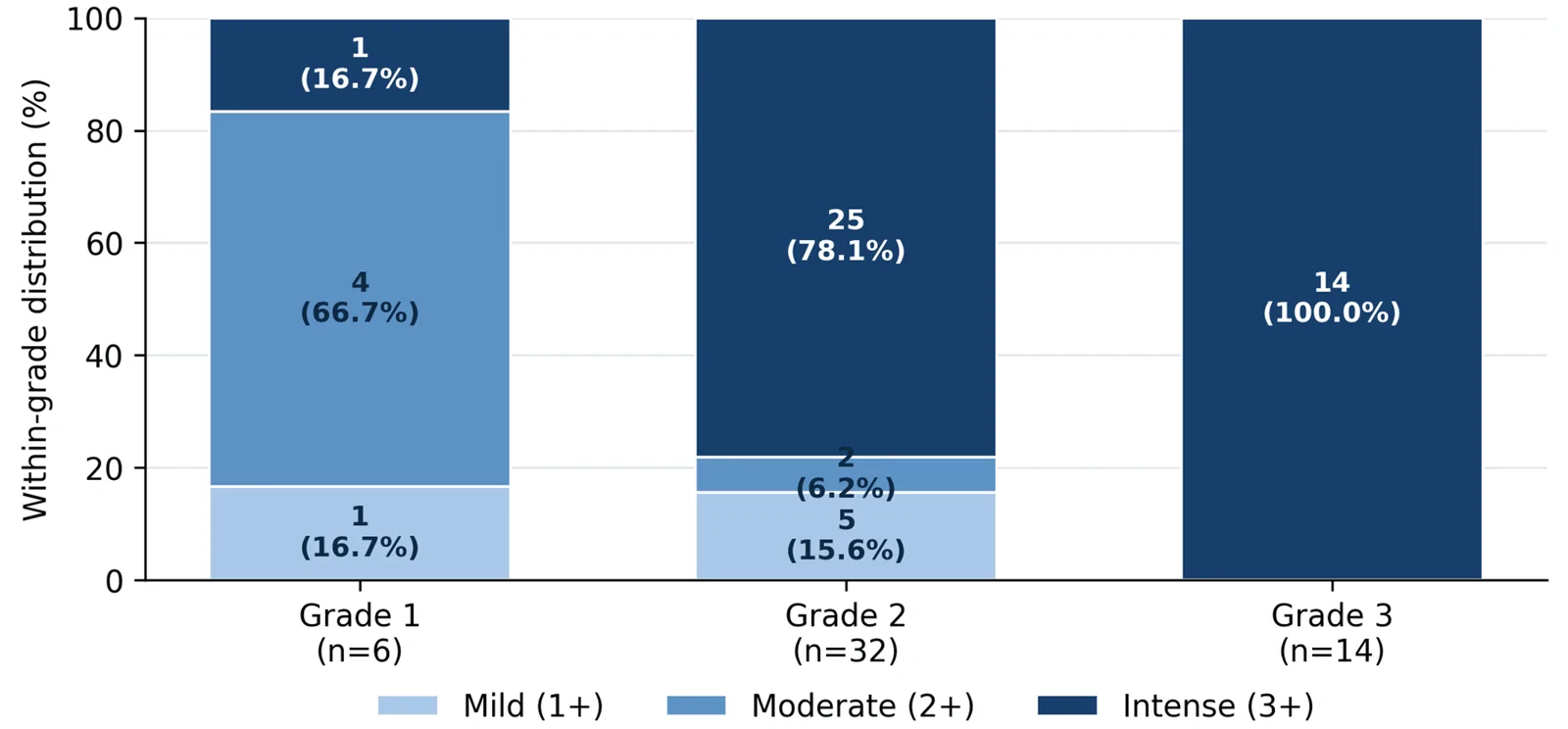
Distribution of proliferating cell nuclear antigen (PCNA) immunoexpression within histological grades The stacked bar chart shows the distribution of mild (1+), moderate (2+), and intense (3+) PCNA immunoexpression within Nottingham histological grades 1, 2, and 3. The respective grade groups included six, 32, and 14 tumors. Each segment is labeled with the number of cases and the corresponding within-grade percentage. The proportion of tumors assigned to the 3+ PCNA immunoexpression category increased across histological grades, and all grade 3 tumors were assigned to the 3+ category. No tumor was assigned to the negative PCNA immunoexpression category.

**Table 4:** Primary and supporting ordinal analyses Monte Carlo procedures used 200,000 samples and 99% confidence intervals; all reported p-values are two-sided. The table emphasizes primary, secondary, and biologically linked analyses rather than exploratory subgroup testing. PCNA: proliferating cell nuclear antigen

Relationship	Method	Effect/statistic	Two-sided p-value
PCNA vs grade	Fisher-Freeman-Halton Monte Carlo	-	<0.001
	Spearman correlation	rho=0.476	<0.001
	Kendall correlation	tau-b=0.438	<0.001
	Jonckheere-Terpstra trend	Z=3.340	<0.001
	Goodman-Kruskal gamma	gamma=0.832	<0.001
	Somers' d (grade dependent)	d=0.519	<0.001
PCNA vs Ki-67	Fisher-Freeman-Halton Monte Carlo	-	<0.001
	Spearman correlation	rho=0.387	0.005
	Kendall correlation	tau-b=0.357	0.005
	Goodman-Kruskal gamma	gamma=0.587	0.016
Ki-67 vs grade	Spearman correlation	rho=0.330	0.017
	Kendall correlation	tau-b=0.316	0.013
	Jonckheere-Terpstra trend	Z=2.491	0.012

Relationship between PCNA and Ki-67

PCNA and Ki-67 immunoexpression categories showed a significant overall association on Fisher-Freeman-Halton Monte Carlo testing (p<0.001; Cramer’s V=0.539). A moderate positive ordinal relationship was observed between the two markers (Spearman rho=0.387, p=0.005; Kendall tau-b=0.357, p=0.005). Goodman-Kruskal gamma was 0.587 (p=0.016). These findings indicate that PCNA and Ki-67 immunoexpression were related but were not interchangeable, as discordant categories were observed in several cases (Tables 4, 5 and Figure 2).

**Table 5:** Cross-tabulation of proliferating cell nuclear antigen (PCNA) and Ki-67 ordinal categories Fisher-Freeman-Halton Monte Carlo p<0.001; Cramer's V=0.539

Ki-67 category	Mild PCNA (1+)	Moderate PCNA (2+)	Intense PCNA (3+)	Total
Negative	1	0	2	3
Mild (1+)	1	6	3	10
Moderate (2+)	3	0	25	28
Intense (3+)	1	0	10	11
Total	6	6	40	52

**Figure 2:**
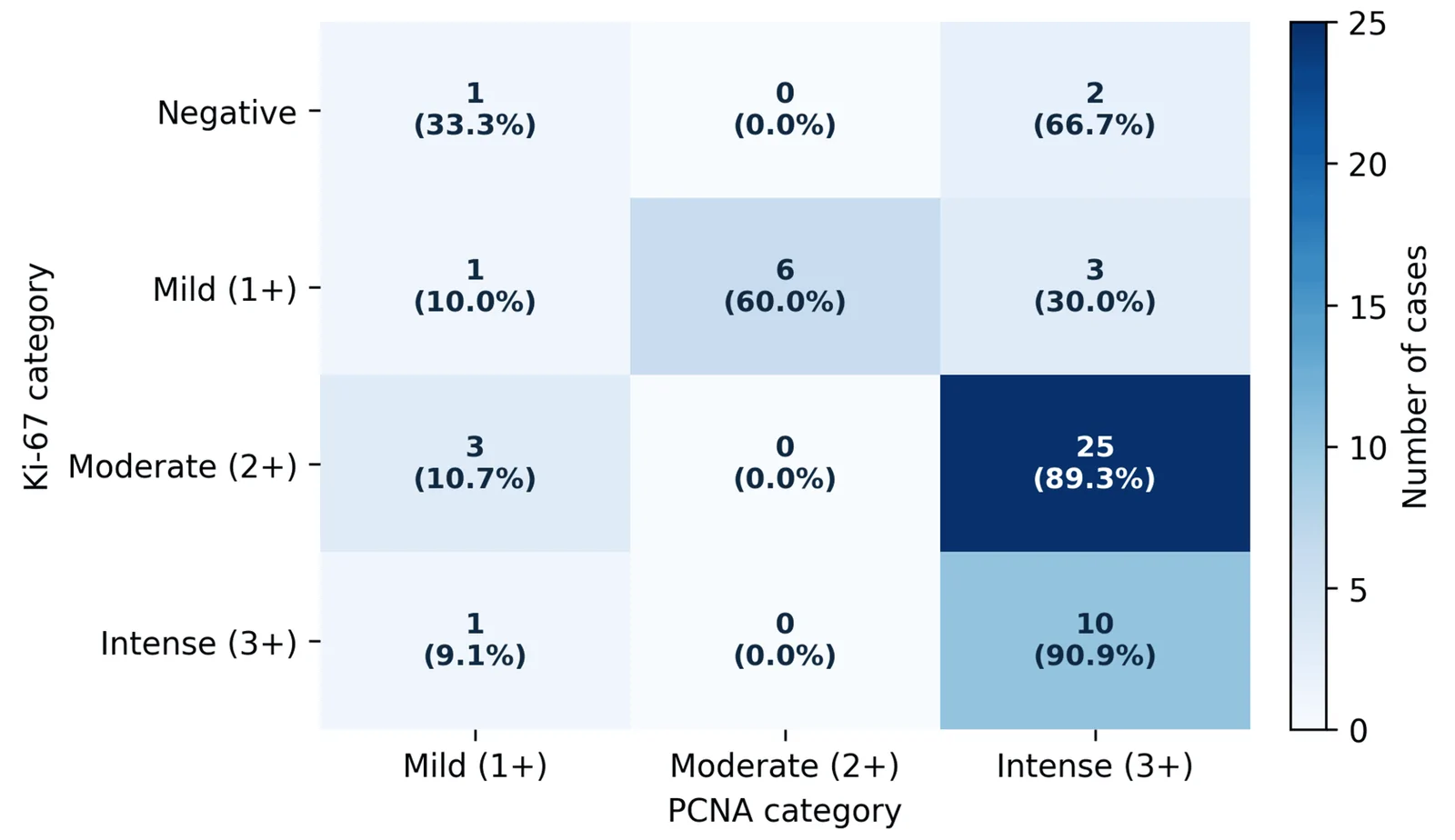
Cross-distribution of proliferating cell nuclear antigen (PCNA) and Ki-67 immunoexpression Cell labels show counts and percentages within each Ki-67 row.

Exploratory associations

Fisher-Freeman-Halton Monte Carlo testing showed no significant association between PCNA category and ER status (p=0.888), PR status (p=0.468), HER2 status (p=0.879), triple-negative phenotype (p=0.888), menopausal status (p=0.420), tumor-size group (p=0.554), or lymph-node group (p=0.981). Stage showed a nominal sparse-table association by asymptotic Pearson testing, but the monotonic PCNA-stage relationship was not significant (Spearman rho=-0.165, p=0.244), and the Fisher-Freeman-Halton Monte Carlo result was p=0.055. Accordingly, stage was not interpreted as being associated with PCNA. The exploratory findings were not used to support causal or prognostic claims. Representative histomorphological and immunohistochemical findings from a grade 3 tumor assigned to the 3+ PCNA immunoexpression category with negative Ki-67 expression are presented in Figure 3. 

**Figure 3:**
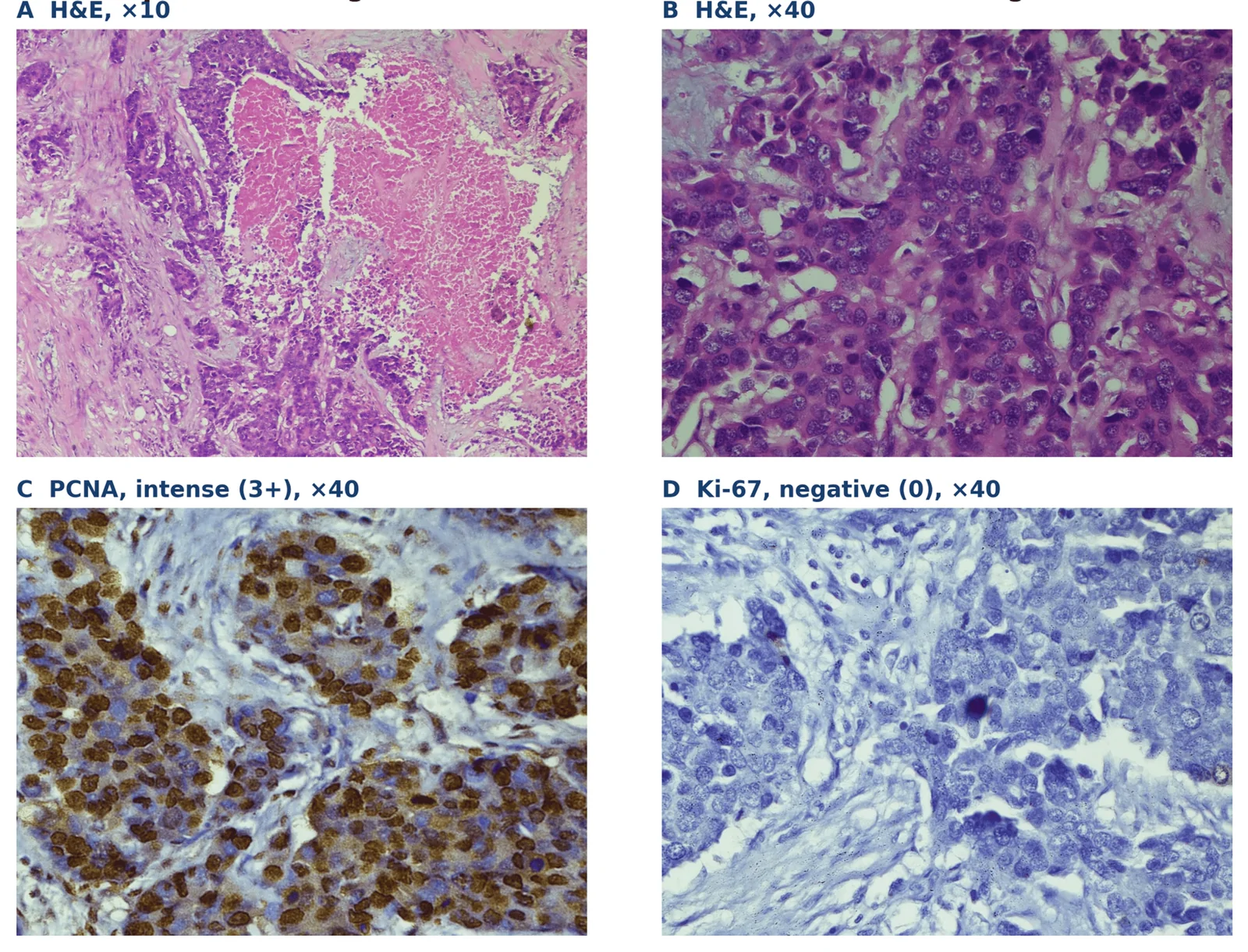
Representative grade 3 invasive ductal carcinoma/no special type image series (A) Tumor arranged in sheets and cords with focal tubule formation (hematoxylin and eosin, x10). (B) Tumor cells showing nuclear hyperchromasia, pleomorphism, an increased nuclear-to-cytoplasmic ratio, and scant cytoplasm (hematoxylin and eosin, x40). (C) Intense nuclear proliferating cell nuclear antigen (PCNA) staining in a tumour assigned to the final 3+ PCNA immunoexpression category based on a composite proportion-plus-intensity score of 6 (×40). (D) Negative Ki-67 nuclear staining recorded as 0 (x40). The series illustrates marker discordance observed in the cohort; images were not used for digital quantification.

## Discussion

Principal findings

This focused analysis demonstrated a consistent positive association between PCNA immunoexpression and histological grade. All grade 3 tumors were assigned to the 3+ PCNA immunoexpression category, compared with 78.1% of grade 2 tumors and 16.7% of grade 1 tumors. The direction of the association was consistent across the selected sparse-table, rank-based, ordered-trend, and ordinal effect-size analyses. PCNA also showed a moderate positive ordinal association with Ki-67, but discordant cases and concentration of PCNA in the 3+ immunoexpression category indicate that the markers are not interchangeable. Because survival and recurrence data were unavailable, the results establish a clinicopathological association rather than prognostic performance.

Comparison with previous evidence

The relationship between PCNA and grade is biologically plausible because histological grade incorporates mitotic activity and morphological features associated with proliferation. Kumar et al. reported associations of PCNA with Ki-67 and tumor grade in mastectomy specimens, while Leonardi et al. and Keshgegian and Cnaan showed that proliferation markers may correlate with histological differentiation but differ in labeling characteristics and prognostic performance [7-9]. Qiu et al. likewise reported relationships among PCNA, Ki-67, and clinical or imaging features [14]. In 205 node-negative breast carcinomas, Aaltomaa et al. found that quantitative PCNA measures were related to grade, S-phase fraction, and mitotic index; some PCNA measures were associated with survival, although tumor size remained important [15]. These studies support a link between PCNA staining and proliferative morphology but used different antibodies, thresholds, and clinical populations, limiting direct numerical comparison.

Haerslev et al. evaluated Ki-67 and PCNA in 487 primary breast carcinomas and found that high expression of both markers was associated with larger tumors, higher grade, and absence of steroid hormone receptors. Neither marker remained independently prognostic after adjustment for classical histopathological variables [16]. This larger study is directionally consistent with the present PCNA-grade finding while reinforcing the distinction between association with aggressive morphology and independent prognostic value.

Recent evidence remains mixed. A 2025 review concluded that PCNA may provide complementary information about aggressive tumor biology, although Ki-67 is more widely used and has a larger clinical evidence base [2]. In contrast, Duran et al. analyzed 302 surgically treated breast cancer patients and found that PCNA was not independently related to the principal clinicopathological variables or overall survival, whereas Ki-67 was associated with receptor status, stage, and survival [10]. Differences from the present study may reflect cohort composition, scoring thresholds, antibody characteristics, specimen handling, and statistical power. The heterogeneous direct literature supports interpreting the current findings as hypothesis-generating rather than as evidence for routine clinical adoption of PCNA.

Recent Ki-67 studies provide biological context for the secondary analyses but do not directly validate PCNA. Rewcastle et al. showed that Ki-67 cutoffs and reproducibility remain method-dependent [17]. Pirić et al. found that high Ki-67 was more frequent with higher grade and nodal metastasis in 106 patients with early ER-positive/HER2-negative disease [18], while Stalhammar et al. demonstrated the potential reproducibility advantages of digital Ki-67 assessment [19]. Sandilya and Mamatha reported significant associations of Ki-67 with grade and tumor size in 55 invasive breast carcinomas [20]. In a 280-case Cureus study, Abubakr et al. observed progressively higher mean Ki-67 values from grade 1 to grade 3 tumors and a strong positive grade correlation [21]. These findings align directionally with the ordered Ki-67-grade trend in the present cohort, but outcome-related conclusions cannot be extended to PCNA without follow-up data.

The PCNA-Ki-67 association supports shared sensitivity to proliferation while also demonstrating biological and technical divergence. PCNA participates in DNA repair and other nucleic-acid processes, and staining may persist in cells that are not actively cycling [5,6]. Ki-67 has a different cell-cycle distribution and remains the more established breast cancer proliferation marker. In real-world data from 4,313 patients, Boyaci et al. found that weighted-global scoring reduced inter-laboratory variability compared with hotspot scoring [22]. Patel and Jawalkar subsequently showed in 53 cases that hotspot scoring produced a higher mean Ki-67 index than weighted-global scoring despite very strong rank correlation between methods [23]. These studies reinforce recommendations for standardized assessment [4] and highlight a central limitation of the present dataset: ordinal categories cannot be recalibrated to a validated quantitative global-scoring method.

The magnitude of the PCNA-Ki-67 relationship in the present cohort also closely resembles earlier direct comparative evidence. Sullivan et al. examined 39 breast carcinomas and reported a weak but statistically significant correlation between PCNA and Ki-67 labeling indices (r=0.38, p<0.005), while PCNA did not correlate with mitotic count [11]. Their correlation estimate is strikingly similar to the present Spearman coefficient of 0.387 and supports interpreting the two stains as related but non-interchangeable proliferation-associated measures. At a broader level, Stuart-Harris et al. systematically reviewed 85 studies involving 32,825 patients and found that proliferation markers, including PCNA, Ki-67, mitotic index, and thymidine-labeling measures, were generally associated with adverse survival outcomes [24]. However, heterogeneity in assays, thresholds, study populations, and adjustment strategies limited conclusions regarding incremental independent prognostic value. This evidence supports the biological relevance of the present association while reinforcing why this cross-sectional study should not make prognostic claims.

Clinical and pathological interpretation

The 3+ PCNA immunoexpression category was common among higher-grade tumors in this cohort and may reflect proliferative morphology when interpreted alongside conventional histology. Nevertheless, 76.9% of all tumors were assigned to the 3+ category, and no tumor was assigned to the negative category, thereby limiting discrimination. The study did not compare PCNA with a quantitative mitotic index, digital Ki-67 labeling index, genomic assay, treatment response, or survival. PCNA may warrant further investigation as a complementary marker in cases showing difficult or discordant Ki-67 interpretation. However, the present findings do not establish that PCNA should influence prognosis, treatment selection, or other patient-care decisions. PCNA should therefore not be proposed as a substitute for Nottingham grade, Ki-67, or established receptor testing on the basis of these data.

Strengths and limitations

Strengths of this study include proportionate stratified random sampling with computer-generated selection within each menopausal stratum, paired PCNA and Ki-67 results from the same tumors, preservation of the ordinal structure of the recorded measurements, correction of triple-negative immunophenotype independently of histological type, and use of Monte Carlo inference and ordered-trend methods appropriate for sparse ordinal data. Agreement among exact, rank-based, trend, and effect-size analyses strengthens confidence that the primary PCNA-grade pattern is not an artifact of a single test.

The study also has important limitations. Although proportionate stratified random sampling preserved the approximate menopausal distribution of the eligible sampling frame and random selection within each stratum reduced investigator-related selection, eligibility required complete essential clinicopathological information and adequate archived tissue for paired PCNA and Ki-67 immunohistochemistry. Exclusion of cases with incomplete information or inadequate tissue may have introduced selection bias. Consequently, the final analytic cohort may not be representative of all 409 breast carcinoma cases identified during the study period. The cohort was small, single-center, entirely female, and dominated by grade 2 and stage 3 disease. PCNA results were concentrated in the 3+ immunoexpression category, and zero-frequency cells precluded stable multivariable modeling. Because the statistical analysis used the final combined immunoexpression categories assigned by the examining pathologist, staining extent and intensity were not analyzed as separate variables. Consequently, the study could not evaluate the independent contributions of these components or apply alternative quantitative scoring thresholds. Immunohistochemical assessment was performed visually by a single pathologist who was not blinded to the available pathological information. Consequently, interobserver reproducibility was not evaluated, and the possibility of observer-related classification variability cannot be excluded. Although the composite PCNA scoring categories were specified in the original study records, their thresholds have not been externally validated against an independent cohort, standardized quantitative measurements, or clinical outcomes. The findings may therefore depend partly on the scoring system and category thresholds applied in this study. Clinical follow-up, including treatment response, recurrence and survival, was unavailable; therefore, the findings demonstrate clinicopathological associations but not prognostic or treatment-predictive performance. As the specimens were collected between 2018 and 2020, validation in a contemporary independent cohort using standardized quantitative scoring is warranted.

## Conclusions

PCNA immunoexpression may serve as a complementary indicator of proliferative activity in invasive breast carcinoma. However, its limited discriminatory range and discordance with Ki-67 prevent it from being considered a substitute for Nottingham histological grade, quantitative Ki-67 assessment, or established receptor testing. Standardized quantitative scoring, assessment of reproducibility, and clinical follow-up will be necessary before PCNA can be considered for routine prognostic evaluation.
